# Evaluating a Venom-Bioinspired Peptide, NOR-1202, as an Antiepileptic Treatment in Male Mice Models

**DOI:** 10.3390/toxins16080342

**Published:** 2024-08-05

**Authors:** Maria Varela Torres Quintanilha, Giovanna de Azevedo Mello Gobbo, Gabriela Beserra Pinheiro, Adolfo Carlos Barros de Souza, Luana Cristina Camargo, Marcia Renata Mortari

**Affiliations:** Laboratory of Neuropharmacology, Department of Physiological Sciences, Institute of Biological Sciences, University of Brasília, Brasília 70910-900, Brazil; mariavtq@gmail.com (M.V.T.Q.); giovannagobbo@gmail.com (G.d.A.M.G.); gabrielapinheiro.unb@gmail.com (G.B.P.); adolfo_quimica@hotmail.com (A.C.B.d.S.); mmortari@unb.br (M.R.M.)

**Keywords:** temporal lobe epilepsy (TLE), antiepileptic drugs, occidentalin-1202, pilocarpine, kainic acid (KA), wasp venom, *Polybia occidentalis*

## Abstract

Epilepsy, a neurological disorder characterized by excessive neuronal activity and synchronized electrical discharges, ranks among the most prevalent global neurological conditions. Despite common use, antiepileptic drugs often result in adverse effects and lack effectiveness in controlling seizures in temporal lobe epilepsy (TLE) patients. Recent research explored the potential of occidentalin-1202, a peptide inspired by *Polybia occidentalis* venom, in safeguarding Wistar rats from chemically induced seizures. The present study evaluated the new analog from occidentalin-1202 named NOR-1202 using acute and chronic pilocarpine-induced models and an acute kainic acid (KA) male mice model. NOR-1202 was administered through the intracerebroventricular (i.c.v.), subcutaneous, or intraperitoneal routes, with stereotaxic procedures for the i.c.v. injection. In the acute pilocarpine-induced model, NOR-1202 (i.c.v.) protected against generalized seizures and mortality but lacked systemic antiepileptic activity. In the KA model, it did not prevent generalized seizures but improved survival. In the chronic TLE model, NOR-1202′s ED_50_ did not differ significantly from the epileptic or healthy groups regarding time spent in spontaneous recurrent seizures during the five-day treatment. However, the NOR-1202 group exhibited more seizures than the healthy group on the second day of treatment. In summary, NOR-1202 exhibits antiepileptic effects against chemoconvulsant-induced seizures, but no effect was observed when administered systemically.

## 1. Introduction

Epilepsy is a multicomplex disorder with different causes that lead to neuronal hyperactivation [1]. The most common type of epilepsy is temporal lobe epilepsy (TLE), which occurs mainly in teenagers and adults. In TLE, it is more common to undergo impaired awareness and behavior arrest seizures. In addition to seizures, mood, psychotic, and anxiety disorders are commonly associated with TLE [2,3]. Causes, varying from the environmental to the genetic, are attributed to the development of epilepsy [1].

To develop new drugs for TLE, preclinical mouse models are essential and offer several advantages due to their similarities with TLE in humans. Among the standard models, chemoconvulsant-induced models can be used for both acute and chronic investigations. Kainic acid (KA) serves as a model for temporal lobe epilepsy, inducing excitatory activity by activating glutamate receptors. KA-induced epilepsy leads to structural alterations in the hippocampus, abnormal neurogenesis, and increased neuroinflammation [4,5]. Additionally, pilocarpine, a chemoconvulsant, induces seizures through its mechanism of action as a cholinergic muscarinic agonist. In chronic models, pilocarpine induces *status epilepticus* (SE) after administration [6]. Unlike the acute model, the chronic epileptic model closely resembles the condition in humans, as mice develop recurrent spontaneous seizures after SE is induced, mimicking TLE. The TLE model in mice is also challenging to treat, paralleling the difficulties seen in human TLE treatment.

TLE patients are known to be drug-resistant, which makes the treatment limited. Antiepileptic drugs (AEDs) mainly act by increasing inhibition and/or decreasing excitation. Most AEDs act on voltage-gated channels and on γ-aminobutyric acid (GABA) receptors [7,8]. Regarding potential treatments for TLE, compounds from animal venom have been tested. Those compounds are potential pharmacological tools due to their selectivity toward the central nervous system (CNS), which provides high efficacy with low toxicity [9]. Bioinspired wasp venom peptides have been studied for many years by our group, with interesting results. A peptide bioinspired by venom of the wasp *Polybia occidentalis* was isolated. Occidentalin-1202 blocked KA- and pentylenetetrazole (PTZ)-induced seizures with low toxicity effects by i.p. administration. This peptide decreases neuron activation, demonstrating a potential neuroprotective effect. Also, in silico studies showed that occidentalin-1202 blocks the kainate receptor [10]. Here, we evaluated an occidentalin-1202 analog, NOR-1202, which had amino acid modification to prevent degradation and increase membrane permeability, in a KA- and pilocarpine-induced epileptic model. Moreover, we also evaluated NOR-1202′s efficacy in the pilocarpine-induced TLE model.

## 2. Results

### 2.1. NOR-1202 Prevented Death in KA-Induced Acute Model

**Figure 1:**
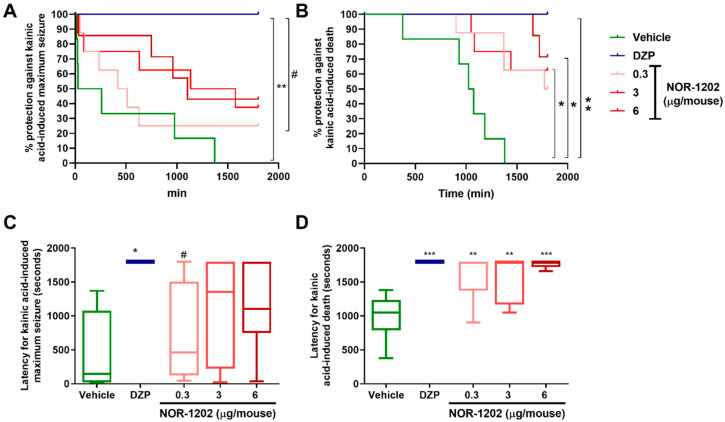
NOR-1202 efficacy in acute kainic acid (KA)-induced seizure via intracerebroventricular (i.c.v.). The effect of NOR-1202 in the percentage against maximum KA-induced seizure (**A**) and the death percentage (**B**). The effect of NOR-1202 on the maximum seizure (**C**) and death latency induced by KA (**D**) (*n* = 8/group). Animals not having maximum seizure but also surviving was considered the maximum time of the test (1800 s). Chi-square and Fisher post hoc were used to calculate the survival curve (**A**,**B**). One-way ANOVA and Tukey post hoc were used (**C**,**D**). *: *p* < 0.05, **: *p* < 0.01 and ***: *p* < 0.001, means significantly different from vehicle group. #: *p* < 0.05, means significantly different from diazepam (DPZ) group.

Considering the protection against generalized seizure (score 7) induced by KA, NOR-1202 in the doses of 6, 3, and 0.3 µg/mouse, infused by i.c.v., protected 42.8%, 37.5%, and 25% of animals, respectively (Figure 1A). NOR-1202 was not different compared with the vehicle group, while diazepam (DZP) showed 100% protection during the 30 min evaluation time, and it was different compared with the vehicle group (Chi-square = 12.45, 4; *p* = 0.0022; Figure 1A). Considering the latency for the first maximum seizure, no differences among the NOR-1202 doses and the vehicle group were observed. Moreover, the 0.3 μg/mouse dose was different compared with the DZP group (F = 3.676; *p* = 0.0154; Figure 1C).

Interestingly, NOR-1202 in doses of 3 and 6 μg/mouse protected 62.5% (*p* = 0.0310) and 71.72% (*p* = 0.0210) of the animals from death, respectively, when compared with the vehicle (Chi-square = 12.49, 4; Figure 1B), while the 0.3 μg/mouse dose protected 50% of the animals (Figure 1B). Similarly to the previous results, DZP prevented death of all the animals (100%) and was different from the vehicle group (*p* < 0.0001, Figure 1B). Considering the latency to death, the NOR-1202 doses showed increased latency compared with the vehicle group (F = 8.363; *p* < 0.01; and *p* < 0.001 for 6 mg/kg; Figure 1D).

### 2.2. NOR-1202 Prevented Deaths in Pilocarpine-Induced Seizures, Acute Model

**Figure 2:**
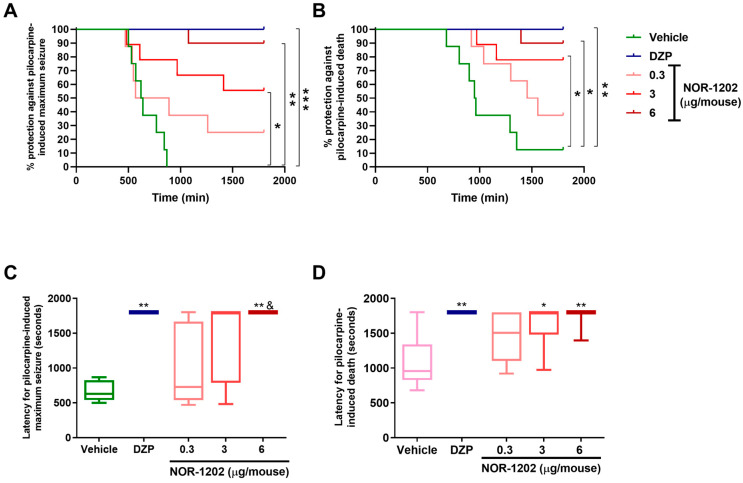
NOR-1202 efficacy in acute pilocarpine-induced seizure via intracerebroventricular (i.c.v.) route. The effect of NOR-1202 in the percentage against maximum pilocarpine-induced seizure (**A**) and death (**B**). The effect of NOR-1202 in the maximum seizure latency (**C**) and death latency induced by pilocarpine (**D**) (*n* = 8/group). Chi-square and Fisher post hoc were used to calculate the survival curve (**A**,**B**). Kruskal–Wallis and Dunn’s post hoc were used (**C**,**D**). *: *p* < 0.05, **: *p* < 0.01, and ***: *p* < 0.001; means significantly different from vehicle group. &: *p* < 0.05, means significantly different from the dose of 0.3 μg/mouse.

Considering the protection against maximum seizure (Scale 7) induced by pilocarpine, NOR-1202 infused by the i.c.v. route was effective compared with the vehicle group as well as DZP (Chi-square = 20.66, 4; *p* = 0.0002; Figure 2A). The higher NOR-1202 dose (6 mg/kg) protected 80% of animals against maximum seizure (*p* = 0.0011). Moreover, the intermediate dose (3 μg/mouse) protected 55.5% (*p* = 0.0294), and the lower dose (0.3 μg/mouse) protected 25% of the animals (Figure 2A). Considering latency for the first maximum seizure, the higher dose of NOR-1202 increased latency compared with the vehicle group as well as compared with DZP (Figure 2C).

Interestingly, NOR-1202 prevented death in doses of 3 and 6 μg/mouse compared with the vehicle (Chi-square = 21.52, 4; Figure 2B). DZP prevented death in all animals (100%; *p* = 0.0014), while the higher dose of the NOR-1202 protected 90% (*p* = 0.0029), the intermediate dose protected 77% (*p* = 0.0152), and the lower dose protected 25% of the animals. Taking the latency for death, the higher and intermediate doses of NOR-1202 showed increased latency compared with the vehicle group, similar to DZP treatment (F = 20.83; *p* < 0.01 and *p* < 0.05, respectively; Figure 2D).

**Figure 3:**
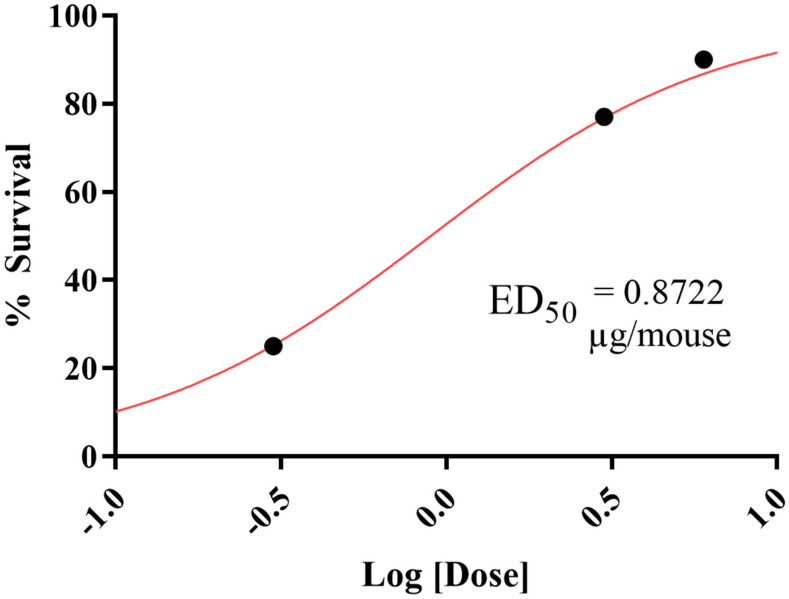
Dose–response curve of NOR-1202. Percentage survival after pilocarpine-induced seizure (R^2^ = 0.9978). The ED_50_ value was calculated by sigmoidal non-linear regression.

Since NOR-1202 showed antiepileptic activity in the pilocarpine-induced seizure acute model via i.c.v., we can estimate that the median effective dose (ED_50_) is around 0.8722 μg/mouse and the confidence interval is 0.3066–2.481 μg/mouse (R^2^ = 0.9978) (Figure 3).

**Figure 4:**
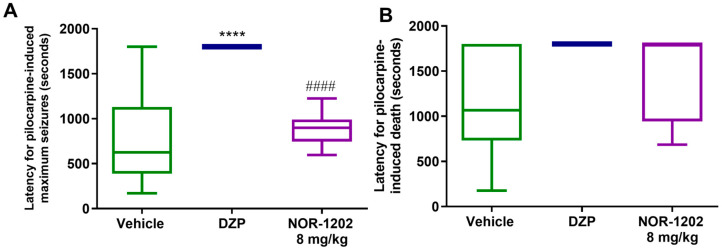
NOR-1202 efficacy in acute pilocarpine-induced seizure via the intraperitoneal (i.p.) route. The effect of NOR-1202 on the maximum seizure induced by pilocarpine (**A**) and on death induced by pilocarpine (**B**) (*n* = 8/group). One-way ANOVA and Tukey post hoc were used. ****: *p* < 0.0001, means significantly different from the vehicle group. ####: *p* < 0.0001, means significantly different from the diazepam (DPZ) group.

**Figure 5:**
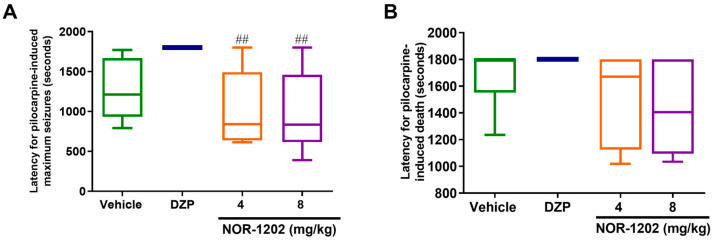
NOR-1202 efficacy in acute pilocarpine-induced seizure via subcutaneous (s.c.) route. The effect of NOR-1202 in the maximum seizure (**A**) and death induced by pilocarpine (**B**) (*n* = 8/group). One-way ANOVA and Tukey post hoc were used. ##: *p* < 0.01, means significantly different from the diazepam (DPZ) group.

When NOR-1202 was administered i.p., no protection was observed regarding death and maximum seizure (Figure 4A,B). Similar results were observed when NOR-1202 was administered by the s.c. route (Figure 5A,B).

### 2.3. NOR-1202 Effect in Pilocarpine-Induced Seizures, Chronic Model

**Figure 6:**
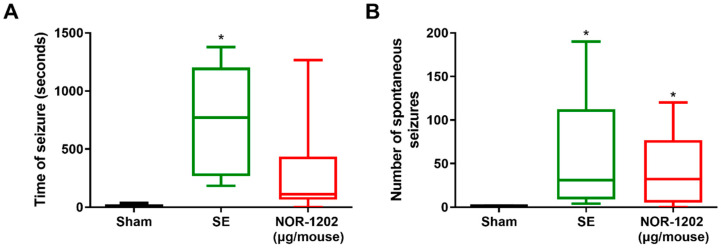
NOR-1202 efficacy in the chronic epileptic model. The treatment effect of NOR-1202 regarding the time (**A**) and number of seizures in a chronic pilocarpine-induced model (**B**) (*n* = 5–8/group). One-way ANOVA and post hoc Tukey were used in (**A**) and Kruskal–Wallis and post hoc Dunn’s were used in (**B**). *: *p* < 0.05, means significantly different from sham group.

In the pilocarpine-induced chronic model, the total time and number of SEs were evaluated. The epileptic group showed an increase in total time of SE compared with the vehicle group (F = 4.501; *p* = 0.0310). No difference was observed in NOR-1202 compared with the vehicle and epileptic groups (Figure 6A). This might indicate that NOR-1202 could have a slight effect in enhancing the seizure time. NOR-1202 had no effect in decreasing the number of SEs in the pilocarpine-induced chronic model (Figure 6B).

## 3. Discussion

In this work, we evaluated the efficacy of an occidentalin-1202 analog to prevent seizures in two chemoconvulsant-induced models. In the acute model, NOR-1202 was able to prevent death in the pilocarpine- and KA-induced tests. Moreover, NOR-1202 also prevented maximum seizure in the pilocarpine-induced model. Those results occurred when NOR-1202 was infused by the i.c.v. route, but these promising results did not occur when NOR-1202 was administered via i.p. and s.c. In the TLE chronic model, no difference was observed comparing NOR-1202 treatment and sham regarding the total time of seizure, although a difference was observed regarding the number of seizures.

Among the AEDs that are used in TLE therapy, benzodiazepine showed efficacy both in humans and in the chronic TLE model. Benzodiazepines (DZP) are sedative drugs that increase γ-aminobutyric acid (GABA) activity, producing a potent inhibition of the central nervous system [11]. In some developed countries, DZP is still used to treat SE. Moreover, DZP has demonstrated neuroprotective effects. Therefore, for the purpose of comparison, we selected this treatment as the control [12,13,14]. As with all sedative drugs, DZP has several side effects, like cognitive deficits, motor impairment, and dependence (for review, [15]). In this work, DZP protected all mice against maximum seizures and death induced by both the KA and pilocarpine acute models. DZP and other AEDs have good pharmacokinetic properties in parenteral administration; therefore, new therapeutic compounds need to also have good systemic effect [8]. In this study, NOR-1202 did not show promising results systemically. Intranasal administration could be promising for peptides due to its enhanced bioavailability to the CNS [8,16]. NOR-1202 was not effective in the TLE model, probably due to the chosen dose of NOR-1202 of 0.87 µg/animal (ED_50_). The ED_50_ of the peptide may have been a low dose, not achieving a strong effect against the CREs, as the peptide seems to have an antiepileptic effect. Moreover, TLE is a condition with many pharmacoresistant patients, and this treatment difficulty is also reproduced in the animal model of TLE [17,18].

Substitution of methionine with norleucine is planned to improve cell membrane penetration and increase the peptide stability [19,20,21,22]. Methionine residues undergo oxidation in biological systems, which might decrease the peptide function and stability [23,24]. Modifications of the vasoactive intestinal peptide (VIP), known as stearyl–norleucine–VIP (SNV), showed improved blood–brain barrier penetration and potency [25]. SNV has a neuroprotective effect against amyotrophic lateral sclerosis and developmental deficiencies [26,27]. In addition, SNV has anti-inflammatory properties [28]. Contrary to what was expected, NOR-1202 did not have good efficacy when administered systemically. NOR-1202 seems to have less effect systemically when compared with occidentalin-1202 [10]. Therefore, it seems that methionine has an essential role in occidentalin-1202 and its analog concerning its pharmacokinetic properties.

Other peptides isolated from wasp venom, as well as its bioinspired peptide, have been demonstrated to have efficacy against chemoconvulsant-induced seizure models. Lopes and co-authors [29] reported that a distinct peptide isolated from *Chartergellus communis* protected against maximum seizure in the pilocarpine and PTZ models. The peptide chartegellus-CP1 showed similar results to NOR-1202 in the pilocarpine acute model via i.c.v. administration with a lower dose (1.5 µg/mouse) [29]. Another peptide, exendin-4, showed an improved KA-induced model survival rate like the NOR-1202 results [30].

Occidentalin-1202 decreased latency time for seizure onset in KA-induced seizures when injected i.p. in all tested doses (4, 2.5, and 1 mg/kg) and via i.c.v. This effect was also observed in the PTZ-induced seizures. Here, NOR-1202 did not decrease the latency time in the KA-induced model via i.c.v., although it was able to decrease the latency time to death. Occidentalin-1202 also had a protective effect in a chronic model of epilepsy in mice via i.c.v. (0.10 µg/animal/day). NOR-1202 was evaluated in the same model and showed no significant effect regarding the total time of seizures. Taking these results, we can assume that methionine amino acid residue is essential to occidentalin-1202 efficacy. It is important to notice that the current study exclusively involved male mice since previous studies of occidentalin-1202 were also evaluated in males. To ensure a comprehensive understanding of therapy efficacy, it is important to evaluate the treatment in females as well. Further experiments are also necessary in order to demonstrate the lack of toxicity of NOR-1202, although occidentalin-1202 has already been shown not to induce motor and cognitive deficits [10].

## 4. Conclusions

Taken together, the NOR-1202 peptide modified from occidentalin-1202 was not as effective as expected, which further supports the hypothesis that the substitution of methionine with norleucine was not beneficial. In summary, NOR-1202 had a protective effect when administered via i.c.v., but this effect was lost when infused i.p. and s.c., probably due to systemic degradation and/or inability to penetrate the BBB. Nevertheless, these results may clarify the roles of amino acids in the peptide sequence and which substitutions could be essential to the compound’s efficacy.

## 5. Materials and Methods

### 5.1. Peptides

NOR-1202 (Glu-Gln-Tyr-Nle-Val-Ala-Phe-Trp-Nle-NH2) was synthesized by AminoTech LTDA (Sorocaba, SP, Brazil) based on the occidentalin-1202 sequence (Glu-Gln-Tyr-Met-Val-Ala-Phe-Trp-Met-NH2), for which the modification was the substitution of methionine with norleucine.

### 5.2. Animals

Male Swiss mice (*Mus musculus*) weighing 18–30 g were used. They were housed in the animal facility of the Biology Institute at UnB in a 12/12 cycle under controlled temperature and humidity, where water and food were given *ad libitum*. All experiments were performed based on the Arouca law and were approved by the CONCEA (Protocol 17/2017).

### 5.3. Neurosurgery

For i.c.v. administration, a cannula was implanted in the right lateral ventricle. For the procedure, animals were placed under anesthesia (ketamine, Ceva, 75 mg/kg i.p.; xylazine, Ceva, 15 mg/kg i.p., Barueri, SP, Brazil) and then placed in a stereotaxic device (Insight Equipamentos^®^, Ribeirão Preto, SP, Brazil). For the cannula implantation in the lateral ventricle, the coordinates were AP: −0.2 mm; ML: −1.0 mm; DV: −2.3 mm, from Bregma, according to Paxinos and Franklin Atlas [31]. Then, the scalp was closed with polymerized acrylic (Dentbras^®^ Brasília, DF, Brazil) and the cannula was sealed. Topic antibacterial cream (neomycin sulfate and bacitracin; 5 mg/g and 250 UL/g; Medley, Campinas, SP, Brazil) was applied to prevent infection. After the surgical procedure, the mice recovery was evaluated for 5–7 days. At the conclusion of the experiment, confirmation of cannula implantation was conducted, and animals with incorrectly positioned cannulas were excluded from the analysis. Based on this criterion, in the acute epileptic KA-induced model experiment, two mice from the vehicle group and two mice from the DZP group, and in the acute epileptic pilocarpine-induced model experiment, one mouse from the diazepam group, were excluded from further analysis.

### 5.4. Epilepsy Model

#### 5.4.1. Acute Epileptic Induced Model

**Figure 7:**
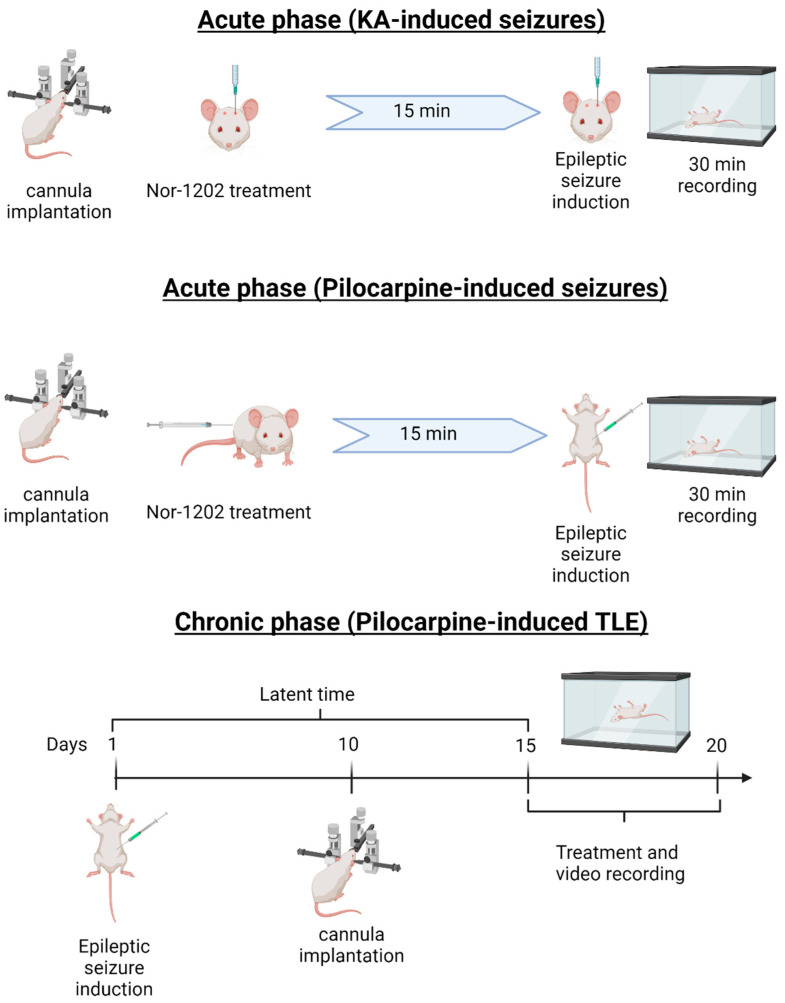
Illustrative schematic of the epileptic models’ methodology. For acute models, a cannula was first implanted for NOR-1202 i.c.v. administration. For the kainic acid (KA)-induced model, NOR-1202 was infused i.c.v. 15 min before KA administration. Then, seizure time and scale were evaluated in a 30 min recording. For the pilocarpine-induced model, NOR-1202 was infused i.c.v., i.p., and s.c. 15 min before pilocarpine administration. Then, seizure time and scale were evaluated in a 30 min recording. In the temporal lobe epileptic model (TLE), pilocarpine was administered on day 1 for status epilepticus induction; then, after 3 h, mice were rescued with thiopental and atropine. On the 10th day, a cannula was implanted for NOR-1202 administration. On the 15th day, the last day of the latent phase, mice were recorded for five consecutive days. Created with BioRender.com (Accessed: 25 February 2024).

All compounds were infused via i.c.v. through the cannula with microsyringe (Gastight 10 μL, Model 1701 N SYR, Hamilton^®^, Reno, NV, USA) in an infused pump (BI-2008, AVS Projetos, São Carlos, SP, Brazil) at 1 μL/min. Three doses of NOR-1202 (6, 3, and 0.3 μg/mouse) or vehicle (0.9% saline and 10% DMSO; 1 μL) were infused in mice; then, after 15 min, KA was also infused via i.c.v. (Figure 7) 1.25 µg/animal) The positive control was treated with DZP (Compaz^®^; 4 mg/kg, Cristália, Itapira, SP, Brazil) via i.p. 30 min before KA infusion.

For the pilocarpine acute model, the treatment protocol was used as described above (Figure 1). Pilocarpine (250 mg/kg) was injected i.p. and methylscopolamine (4 mg/kg; Boehringer Ingelheim, Ingelheim am Rhein, Germany) was injected i.p. to prevent pilocarpine side effects and reduce deaths [32]. In addition to i.c.v. infusion, NOR-1202 was also injected s.c. (8 and 4 mg/kg) and i.p. (8 mg/kg) 30 min prior to pilocarpine injection (Table 1).

#### 5.4.2. Epileptic Screening

To evaluate NOR-1202 efficacy in preventing epileptic seizures, two criteria were evaluated. First was the latency time for the first maximum seizure establishment, and then the protection percentage against the maximum seizure. Second was the latency time for death and the protection percentage against death. Animals that did not have maximum seizure but also survived were considered as the maximum time of the test (1800 s). The classification of the seizures was evaluated based on the Racine index [33], modified by Shibley and Smith (2002) [34] for the pilocarpine model (Table 2). For the KA-induced epileptic seizure model, the seizure evaluation was based on the Racine index (1971), modified by Pinel and Rovner (1978) [35] (Table 3).

#### 5.4.3. Temporal Lobe Epilepsy (TLE) Model

To evaluate NOR-1202 efficacy in preventing spontaneous and recurrent seizures, the treatment occurred in a pilocarpine-induced TLE model (Figure 7). For that, *SE* was induced in a similar way to the pilocarpine-induced acute phase (Table 4). Mice then presented seizures, and, after 180 min, they were rescued using thiopental (50 mg/kg, i.p.) and atropine (0.1 mg/animal, i.p.; Hypofarma, Ribeirão das Neves, MG, Brazil). On the 10th day, a cannula was implanted as mentioned above. On the 15th day, the treatment started for 5 days. During the treatment, all mice were monitored for 10 h/day. For the behavior analysis, the evaluation was performed by a blind experimenter. The analysis was divided into immediate, as the first 5 h after the treatment, and delayed, from 5 to 10 h.

#### 5.4.4. Analyses

All statistical analyses were performed in the software GraphPad Prism^®^ 7.0 (San Diego, CA, USA). First, normal distribution was evaluated by Shapiro–Wilk, Kolmogorov–Smirnov, and D’Agostino and Pearson. Then, the data were analyzed by one-way ANOVA or Kruskal–Wallis. Chi-square and Fisher’s post hoc were used for analyzing survival and seizure percentages. The ED_50_ was calculated by sigmoidal non-linear regression.

## Figures and Tables

**Table 1:** Experimental group summary used in the acute phase. Abbreviation: dimethyl sulfoxide (DMSO); diazepam (DZP); kainic acid (KA); intraperitoneal (i.p.); subcutaneous (s.c.); intracerebroventricular (i.c.v.).

Group	Treatment	Chemical Induction
Vehicle (*n* = 8)	0.9% saline + 10%DMSO	
DZP (*n* = 8)	4 mg/kg, i.p.	KA (i.c.v.)
NOR-1202 (*n* = 8/group)	6 μg/mouse, i.c.v.	
	3 μg/mouse, i.c.v.	
	0.3 μg/mouse, i.c.v.	
DZP (*n* = 8)	4 mg/kg, i.p.	Pilocarpine (i.p.)
NOR-1202 (*n* = 8/group)	6 μg/mouse i.c.v.	
	3 μg/mouse i.c.v.	
	0.3 μg/mouse i.c.v.	
	4 mg/kg i.p.	
	8 mg/kg i.p.	
	4 mg/kg s.c.	

**Table 2:** Evaluation of pilocarpine-induced limbic seizures based on the Racine index (1972) [33], modified by Shibley and Smith (2002) [34].

Scale	Evaluation
1 and 2	Facial automatism, tail stiffness, short-term tremors, freezing before previous behaviors
3	Low-intensity tonic-clonic seizures, unilateral myoclonic of limbs
4	Bilateral myoclonic of limbs
5	General seizures

**Table 3:** Evaluation of KA-induced epileptic seizures based on the Racine index (1971), modified by Pinel and Rovner (1978) [35].

Scale	Evaluation
1 and 2	Orofacial movement and myoclonic of head
3	Myoclonic of anterior paws
4 and 5	Elevation and fall
6	All of the above in sequence
7	Vocalization, rolling and repeated violent jumps, in addition to a period of hypertonus

**Table 4:** Experimental group summary used in the temporal lobe epilepsy (TLE) model. Abbreviation: dimethyl sulfoxide (DMSO); intraperitoneal (i.p.); intracerebroventricular (i.c.v.); median effective dose (ED_50_).

Group	Treatment	Chemical Induction
Sham (*n* = 5)	0.9% saline + 10%DMSO (1 μL; i.c.v.)	No induction
Epileptic (*n* = 5)	0.9% saline + 10%DMSO (1 μL; i.c.v.)	Pilocarpine (i.p.)
NOR-1202 (*n* = 8)	ED_50_ dose (0.8722 μg/mouse; i.c.v.)	Pilocarpine (i.p.)

## Data Availability

All data are available in this manuscript.
